# Burden of Seasonal Human Coronavirus Infections in Hematopoietic Cell Transplant Recipients

**DOI:** 10.1111/tid.70094

**Published:** 2025-08-21

**Authors:** Fareed Khawaja, Terri Lynn Shigle, Layale Yaghi, May Daher, Jeremy L. Ramdial, Ella Ariza‐Heredia, Ying Jiang, Roy F. Chemaly

**Affiliations:** ^1^ Department of Infectious Diseases, Infection Control, and Employee Health The University of Texas MD Anderson Cancer Center Houston Texas USA; ^2^ Department of Pharmacy Clinical Programs The University of Texas MD Anderson Cancer Center Houston Texas USA; ^3^ Department of Stem Cell Transplantation and Cellular Therapy The University of Texas MD Anderson Cancer Center Houston Texas USA

**Keywords:** coronavirus, hematopoietic cell transplant, outcome, stem cell transplant

## Abstract

**Background:**

Community respiratory viruses, such as seasonal human coronavirus (HCoV), commonly infect hematopoietic cell transplant (HCT) recipients. Recognizing the risk factors and outcomes of HCoV infections in HCT recipients is essential for the future development of potentially lifesaving therapeutics.

**Methods:**

We performed a retrospective review of all HCoV‐infected HCT recipients from September 1, 2015 to August 31, 2017, at our institution. Patients were classified with upper respiratory tract infection (URI) or lower respiratory infection (LRI) based on predefined definitions for respiratory viral infections in HCT recipients. Patient data were collected to identify risk factors for HCoV LRI, and to calculate an immunodeficiency scoring index (ISI). Univariate and multivariate analysis were performed to identify risk factors for LRI.

**Results:**

We identified 164 episodes in 138 HCT recipients (129 URI and 35 LRI) during the study period with an incidence of HCoV of 9%. Overall, 30‐day mortality was 17% and 0%, among patients with HCoV LRI or URI, respectively. On multivariate analysis, low‐albumin, coinfection with multiple respiratory viruses, and an ISI ≥ 5 were independent predictors of LRI and the latter was associated with increased risk of hospital admission, ICU admission, mechanical ventilation, and 30‐day mortality.

**Conclusions:**

We identified unique characteristics that were associated with HCoV LRI in HCT recipients. An ISI ≥ 5 predicted HCoV LRI in HCT recipients.

## Graphical abstract

In this large study, we demonstrated that a high ISI is independently associated with HCoV LRI in HCT recipients. HCoV LRI was associated with several adverse outcomes such as ICU admission, oxygen requirement, and mortality. The ISI potentially may be used to identify high‐risk HCT recipients with HCoV infections.

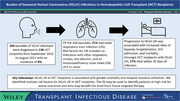

## 1 Introduction

Community respiratory viruses (CRV) are common infectious complications among hematopoietic cell transplant (HCT) recipients and include influenza, parainfluenza virus (PIV), respiratory syncytial virus (RSV), rhinovirus, and human coronavirus (HCoV) [1]. Infections related to these viruses are associated with grave outcomes such as progression from upper respiratory tract infection (URI) to lower respiratory tract infection (LRI), increased airway resistance, increased oxygen use, and mortality [2, 3].

Advances in molecular testing improved the detection of HCoV and other respiratory viruses more consistently [4]. HCoV has been described as a common cause of respiratory viral infection in HCT recipients and patients with hematologic malignancies with a reported seasonal incidence up to 17% [4, 5, 6]. Seasonal HCoV consists of strains such as OC43, NL63, 229E, and HKU1. The risk of HCoV progression from URI to LRI in HCT recipients can occur in up to 30% [4]; however, the mortality rate associated with HCoV LRI in HCT recipients was high, around 50% among allogeneic HCT recipients in one study [6], almost comparable to the reported mortality in HCT recipients with coronavirus disease 2019 (COVID‐19) prior to mass vaccination and access to different treatment options [7, 8]. On the other hand, therapeutic options for patients with HCoV URI or LRI are limited to supportive care as no targeted therapies are available.

Numerous host factors have been described as predictors of poor outcomes in HCT recipients with RSV, influenza, or rhinovirus infections [9, 10, 11, 12]. An immunodeficiency scoring index (ISI) for HCT recipients with RSV infection was derived from host related risk factors for progression to LRI and mortality [9]. A moderate or high ISI correlated with poor clinical outcomes in HCT recipients infected with other respiratory viruses, such as influenza and parainfluenza [13, 14, 15, 16] signifying the possible versatility of this scoring index in HCT recipients. Based on the ISI, guidance for management of RSV‐infected HCT recipients who are at high risk for complications have been proposed [14, 17]. Furthermore, the ISI could be used to risk‐stratify HCT recipients diagnosed with HCoV to predict progression to severe disease and mortality.

Classifying immunocompromised patients who are at high risk for complications and mortality from HCoV infections is imperative for future therapeutic trials. In this study, we report the clinical characteristics and outcomes of HCT recipients with HCoV infections, including progression to LRI and mortality after risk stratification based on the ISI.

## 2 Methods

We performed a retrospective cohort analysis of all consecutive HCT recipients at The University of Texas MD Anderson Cancer Center (Houston, Texas, USA) with laboratory diagnosed HCoV infections between September 1, 2015, and August 31, 2017. We included all adult patients (aged ≥ 18 years), with any type or source of HCT. Baseline characteristics, clinical, laboratory, and outcomes data were collected from the electronic medical records for each episode of HCoV infection. An ISI was calculated for all included patients, using data at the time of HCoV diagnosis (ISI components displayed in Table S1). Based on the ISI score, episodes were stratified as low (0–2), moderate (3–7), or high (> 7) risk for worse outcomes. The primary variables of interest were HCoV URI and HCoV LRI (including possible, probable, and progression). Other variables included hospitalization, length of hopsital stay, intensive care unit (ICU) admission, supplemental oxygen use, mechanical ventilation, and 30‐day mortality. Definitions of the study terminology are depicted in Table 1.

**TABLE 1:** Definitions for study terminology used.

**Terminology**	**Definition**
HCoV episode	The time interval from symptom onset to symptom resolution due to a confirmed HCoV infection (OC43, NL63, 229E, or HKU1 strains). This interval was set at 4 weeks in cases in which symptom resolution was not recorded in the medical record.
HCoV URI	An episode in which a patient is symptomatic (cough, rhinorrhea, or shortness of breath) and HCoV (OC43, NL63, 229E, or HKU1 strains) was detected in the nasopharynx, with no radiologic evidence of LRI. This definition is similar to previous definitions of RSV infection in HCT recipients.
Possible HCoV LRI	An episode in which a patient is symptomatic (cough, rhinorrhea, or shortness of breath) and HCoV (OC43, NL63, 229E, or HKU1 strains) is detected in the nasopharynx, with radiologic evidence of LRI but no bronchoalveolar lavage sampling. This definition is similar to previous definitions of RSV infection in HCT recipients.
Probable HCoV LRI	An episode in which a patient is symptomatic (cough, rhinorrhea, or shortness of breath) and HCoV (OC43, NL63, 229E, or HKU1 strains) was detected in the nasopharynx, with radiologic evidence of LRI and HCoV detection in bronchoalveolar lavage samples, similar to prior studies on HCoV infections in HCT recipients.
Progression to HCoV LRI	An episode in which a symptomatic patient is diagnosed originally with HCoV URI but after 2 days or more, develops new signs of HCoV LRI based on repeat imaging and/or bronchoscopy.
Nosocomial HCoV	HCoV infection that developed at least 72 h after admission to the hospital.
Hypoalbuminemia	Serum albumin of < 3 g/dL.
Lymphopenia	An absolute lymphocyte count of ≤ 200 cells/mL.
Neutropenia	An absolute neutrophil count of ≤ 500 cells/mL in serum.

### 2.1 Statistical Analysis

We used medians with interquartile ranges and frequencies with percentages to describe continuous and categorical data, respectively. Categorical variables were compared using chi‐square or Fisher's exact test when appropriate and continuous variables compared using Wilcoxon rank sum test. For the statistical analysis, HCoV LRI included episodes of possible HCoV LRI, probable HCoV LRI, and progression to HCoV LRI. Multivariate logistic regression analysis was used to identify the independent risk factors for LRI HCoV.

To determine an optimum ISI cutoff, we generated a receiver operating characteristic (ROC) curve using LRI as the outcome of interest. A second multivariate analysis was performed using this cutoff, replacing variables that are components of the ISI. A similar analysis was used to determine risk factors for mortality. All tests were two‐sided tests with a significance level of 0.05. We also compared outcomes, such as oxygen use rates, admission rates, length of hospital and ICU stay, and mechanical ventilation rates using Fisher's exact test or Wilcoxon test when appropriate. The statistical analyses were performed using SAS version 9.3 and JMP Pro Version 15 (SAS Institute Inc., Cary, NC).

### 2.2 Ethical Considerations

This study was approved by the University of Texas MD Anderson's institutional review board, and a waiver of patient's consent was granted. All patients' data were stored in an encrypted file and de‐identified for use in the analysis.

## 3 Results

### 3.1 Cohort Characteristics

We identified 138 HCT recipients with laboratory confirmed HCoV infections resulting in 164 episodes. Overall, 1466 HCTs were performed at our institution during the study period and the incidence of HCoV infections was 9%. Most episodes occurred during the Fall Winter season (Figure S1).

Briefly, most of the patients were White (59%), the most common comorbidities were lung diseases (19%), diabetes mellitus (16%), and heart failure (4%), and 30% of patients had a history of smoking (Table 2). The most common indications for HCT were acute myelogenous leukemia (29%) and acute lymphoblastic leukemia (17%). Most patients underwent allogeneic HCT (77%) from a matched donor (56%), and most had myeloablative conditioning regimens (91%) prior to transplantation. The median number of days from HCT to HCoV infection was 329 days (IQR = 92–798). A large proportion of patients were on immunosuppressive therapy (63%) or steroid therapy (41%) at the time of or within 2 weeks of being infected. Other baseline characteristics are displayed in Table 2.

**TABLE 2:** Baseline characteristics of human coronavirus infections in hematopoietic cell transplant recipients at diagnosis.

**Characteristic**	**Total (*n* = 164)**	**LRI (*n* = 35)**	**URI (*n* = 129)**
Age, years (IQR)	54 (36–63)	47 (33–60)	56 (39–54)
Female sex, *n* (%)	84 (51)	17 (49)	68 (52)
Race or ethnicity, *n* (%)			
White	97 (59)	22 (63)	75 (58)
Hispanic	40 (24)	7 (20)	33 (26)
Black	13 (8)	2 (6)	11 (8)
Asian	7 (4)	2 (6)	5 (4)
Middle Eastern	6 (4)	1 (3)	5 (4)
Other	1 (1)	1 (3)	0 (0)
Prior lung disease, *n* (%)	32 (20)	7 (20)	25 (19)
Prior history of heart failure, *n* (%)	6 (4)	1 (3)	5 (4)
Prior history of diabetes mellitus type 2, *n* (%)	27 (16)	3 (9)	24 (19)
History of smoking, *n* (%)	49 (30)	11 (31)	39 (29)
Underlying malignancy, *n* (%)			
Acute myeloid leukemia	47 (29)	14 (40)	33 (26)
Acute lymphocytic leukemia	28 (17)	6 (17)	22 (17)
Chronic myeloid leukemia	11 (7)	2 (6)	9 (7)
Chronic lymphocytic leukemia	11 (7)	1 (3)	10 (8)
Hodgkin's lymphoma	13 (8)	2 (6)	11 (9)
Non‐Hodgkin's lymphoma	20 (12)	2 (6)	18 (14)
Multiple myeloma	16 (10)	2 (6)	14 (11)
Myelodysplastic syndrome	10 (6)	2 (6)	8 (6)
Other	8 (5)	4 (11)	4 (3)
Remission	144 (88)	31 (89)	113 (88)
Type of HCT, *n* (%)			
Matched related donor	48 (29)	11 (31)	37 (29)
Matched unrelated donor	44 (27)	10 (29)	34 (26)
Haploidentical donor	24 (15)	5 (14)	19 (15)
Mismatched donor	10 (6)	4 (11)	6 (5)
Autologous	38 (23)	5 (14)	33 (26)
Stem cell source, *n* (%)			
Cord blood	13 (8)	4 (11)	9 (7)
Bone marrow	40 (24)	11 (31)	29 (22)
Peripheral blood	111 (67)	20 (57)	91 (71)
Engraftment at time of infection, *n* (%)^*^	157 (96)	31 (89)	126 (98)
Myeloablative conditioning, *n* (%)	149 (91)	31 (89)	118 (91)
Days from transplantation (IQR)^*^	329 (92–798)	141 (72–473)	366 (98–889)
Steroids within 2 weeks, *n* (%)^*^	66 (41)	20 (57)	46 (36)
Immunosuppressive therapy within 2 weeks^a^	103 (63)	27 (76)	76 (59)
Acute GVHD within 2 weeks of diagnosis, *n* (%)	14 (9)	5 (16)	9 (10)
Lungs	0 (0)	0 (0)	0 (0)
Skin	8 (5)	2 (6)	6 (5)
Gastrointestinal	6 (4)	3 (9)	3 (2)
Eyes	0 (0)	0 (0)	0 (0)
Chronic GVHD within 2 weeks of diagnosis, *n* (%)	40 (24)	5 (17)	35 (27)
Lungs	11 (6)	2 (6)	9 (7)
Skin	26 (16)	2 (6)	24 (18)
Gastrointestinal	10 (6)	1 (3)	9 (7)
Eyes	12 (7)	1 (3)	11 (8)
Other^b^	13 (8)	2 (6)	11 (8)
White blood cell count, k/µL (IQR)	5.2 (3.3–8.0)	3.9 (2.5–5.8)	5.6 (3.6–8.4)
ANC, cells/mL (IQR)^*^	3105 (1920–4923)	2950 (1380–4000)	3140 (1920–5130)
ANC < 500 cells/mL (%)^*^	10 (6)	6 (17)	4 (3)
ALC, cells/mL (IQR)	945 (400–1798)	500 (60–1030)	1090 (485–1970)
ALC < 200 cells/mL (%)^*^	18 (11)	10 (29)	8 (6)
Creatinine, mg/dL (IQR)	0.91 (0.7–1.2)	1.05 (0.75–1.3)	0.9 (0.73–1.14)
Albumin, g/dL (IQR)^*^	3.8 (3.5–4.2)	3.5 (3.0–3.8)	4 (3.6–4.2)
Albumin ≤ 3 g/dL, *n* (%)^*^	13 (8)	10 (29)	3 (2)
ISI grade, *n* (%)^*^			
Low^c^	31 (21)	7 (20)	24 (19)
Moderate^c^	119 (73)	21 (60)	98 (76)
High^c^	14 (9)	7 (20)	7 (5)
ISI ≥ 5	46 (28)	19 (54)	27 (21)
Human coronavirus serotype, *n* (%)			
OC43	55 (34)	8 (23)	47 (36)
NL63	46 (28)	10 (29)	36 (28)
229E	43 (26)	14 (40)	29 (22)
HKU1	31 (19)	6 (17)	25 (19)
Coinfection with multiple human coronavirus strains, *n* (%)	11 (7)	3 (9)	8 (6)
Coinfection with other respiratory viruses, *n* (%)^*^	48 (29)	22 (63)	26 (20)
Respiratory syncytial virus	10 (7)	5 (14)	5 (4)
Rhinovirus	17 (10)	6 (17)	11 (9)
Metapneumovirus	7 (4)	3 (9)	4 (3)
Adenovirus	7 (4)	3 (9)	4 (3)
Parainfluenza	9 (5)	5 (14)	4 (3)
Nosocomial infection, *n* (%)^*^	15 (9)	7 (20)	8 (6)

Abbreviations: ALC, absolute lymphocyte count; ANC, absolute neutrophil count; GVHD, graft versus host disease; HCT, hematopoietic cell transplantation; ISI, immunodeficiency scoring index; LRI, lower respiratory tract infection; URI, upper respiratory tract infection.

^a^ Mycophenolate mofetil, alemtuzumab, anti‐thymocyte globulins, rituximab, sirolimus, tacrolimus, pentostatin, cyclosporine, methotrexate, rapamycin, and cyclophosphamide.

^b^ Other GVHD involved the liver (acute) or the joints.

^c^ ISI grades were analyzed as an ordinal variable.

* *p* < 0.05.

The most common HCoV serotype was OC43 (34%), followed by NL63 (28%) and 229E (27%); 11 (7%) episodes included 2 serotypes. Coinfection with a second respiratory virus occurred in 48 (29%) episodes. The most common coinfecting viruses were rhinovirus (10%) and RSV (7%). Other infection‐related characteristics are depicted in Table 2.

### 3.2 Risk Factors for HCoV LRI

Of the 164 episodes of HCoV infections, 129 (79%) and 35 (21%) were at the URI and the LRI stage, respectively. We included six patients who progressed to LRI after initially presenting as URIs in the LRI cohort. On univariate analysis, infections with HCoV prior to engraftment, time from HCT, steroids use within 2 weeks of HCoV infection, neutropenia, lymphopenia, hypoalbuminemia, coinfections with other respiratory viruses, and nosocomially acquired infections were associated with LRI (Table 2). No specific serotype was associated with LRI (Figure S2).

### 3.3 Validation of the ISI

Of the 164 episodes of HCoV infections, 119 (72%) were classified as moderate, 31 (19%) as low, and 14 (9%) as high risk based on the ISI (Table 2). Based on a ROC curve (Figure S3), we determined that an ISI score of ≥ 5 was the optimal cut off value to predict LRI (sensitivity of 54%, specificity of 79%, positive predictive value of 40%, and negative predictive value of 87%). When comparing ISI in patients with LRI or URI; more patients with LRI had high ISI (20% vs. 5%, *p* = 0.0248) (Table 2).

We performed a logistic regression analysis to identify independent predictors of HCoV LRI (Table 3). In model 1, we included the main risk factors for progression to LRI as separate variables and included neutropenia, lymphopenia, use of steroids, age, time from transplant, time from engraftment, use of myeloablative conditioning, respiratory viral coinfections, hypoalbuminemia, and nosocomial infections. Respiratory viral coinfections and hypoalbuminemia were independent predictors of LRI (Table 3, model 1). In model 2, we replaced all ISI components with an ISI cutoff of 5. ISI ≥ 5, hypoalbuminemia, and respiratory viral coinfections were independently associated with LRI (Table 3, model 2).

**TABLE 3:** Independent variables of lower respiratory infections by multivariate logistic regression analysis.

**Model 1**			
**Predictors**	**Adjusted odds ratio**	**95% CI**	***p* value**
Albumin ≤ 3 g/dL	16.44	3.77–71.75	0.0002
Coinfection with other respiratory viruses	6.62	2.74–15.96	< 0.0001

**Model 2**			
**Predictors**	**Adjusted odds ratio**	**95% CI**	***p* value**
Albumin ≤ 3 g/dL	13.31	2.91–60.82	0.0008
Coinfection with respiratory viruses	5.91	2.40–14.57	0.0001
ISI ≥ 5	3.03	1.22–7.53	0.017

*Note*: In model 1, we added to the model ANC < 500 cells/mL, ALC < 200 cells/mL, age > 40 years, myeloablative conditioning regimen, GVHD (acute or chronic), corticosteroids, and hematopoietic cell transplantation. In model 2, these variables above were replaced with the immunodeficiency scoring index ≥ 5.

Abbreviations: 95% CI = 95% confidence interval; ALC: absolute lymphocyte count; ANC: absolute neutrophil count; GVHD: graft versus host disease; ISI: immunodeficiency scoring index.

### 3.4 Outcomes Associated With LRI

**FIGURE 1:**
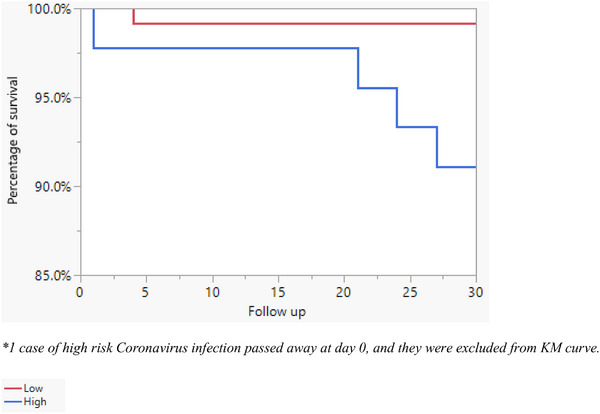
Kaplan–Meier curve demonstrates survival difference between high (score ≥ 5) and low (< 5) immunodeficiency scoring index in hematopoietic cell transplant recipients with human coronavirus infections (cumulative probability as *Y* axis, log rank = 0.0021). ^*^ One case of high risk coronavirus infection passed away at Day 0, and they were excluded from KM curve.

Only six patients (4%) died within 30 days of HCoV infections (Table 4, Figure 1). All deaths were due to respiratory failure in HCT recipients who had HCoV LRI. Due to the low number of deaths, further analysis was not possible. In addition, HCoV LRI was associated with worse outcomes in our HCT recipients than in patients with HCoV URI. These outcomes included higher rates of hypoxia that required supplemental oxygen use (30% vs. 3%, *p* < 0.0001), admission rates (80% vs. 21%, *p* < 0.0001), ICU admission (37% vs. 2%, *p* < 0.0001), longer ICU stay (6 days vs. 1 day, *p* = 0.0024), mechanical ventilation (23% vs. 1%, *p* < 0.0001), and mortality (17% vs. 0%, *p* < 0.0001) (Table 4).

**TABLE 4:** Comparison of outcomes stratified by human coronavirus lower respiratory tract infection versus upper respiratory tract infection.

**Variable**	**Total (*n* = 164)**	**LRI (*n* = 35)**	**URI (*n* = 129)**	***p* value**
Hypoxia at diagnosis, *n* (%)	14 (8)	10 (30)	4 (3)	< 0.001
Admission rate, *n* (%)	55 (34)	28 (80)	27 (21)	< 0.001
Median LOS (IQR), days	11 (5–28)	13 (6–31)	10 (5–25)	0.14
ICU admissions, *n* (%)	16 (29)	13 (35)	3 (2)	< 0.001
Median ICU LOS (IQR), days	4.5 (1–11)	6 (2–12)	1 (1–1)	0.0025
Mechanical ventilation, *n* (%)	9 (5)	8 (23)	1 (1)	< 0.001
30‐day mortality, *n* (%)	6 (4)	6 (17)	0 (0)	< 0.001

Abbreviations: ICU, intensive care unit; IQR, interquartile range; LOS, length of stay; LRI, lower respiratory tract infection; URI, upper respiratory tract infection.

## 4 Discussion

In this large cohort study, we demonstrated that a high ISI (> or equal to 5) is independently associated with HCoV LRI in HCT recipients. We also identified independent predictors of HCoV LRI such as low albumin at the time of infection, and coinfections with other respiratory viruses. In addition, HCoV LRI was associated with several adverse outcomes such as ICU admission, oxygen requirement, mechanical ventilation, and mortality. The utility of the ISI in identifying high‐risk HCT recipients with HCoV infections that may potentially benefit from targeted therapy need to be determined in future clinical trials.

Multiple risk factors for progression to LRI in HCoV‐infected HCT recipients were identified in prior studies [4, 18]. Pinana et al. reported that lymphopenia or ALC < 500 cell/mL, active graft‐versus‐host disease, HCoV infection occurring after the first year of transplant, and fever at time of presentation were independent risk factors for HCoV LRI in allogeneic HCT recipients [18]. Ogimi et al. recently reported that male gender, hypoalbuminemia (< 3 g/dL), hyperglycemia (> 150 mg/dL), presence of respiratory co‐pathogens and high ISI (7–12) as risk factors for occurrence of LRI [16]. In comparison, we identified similar but fewer risk factors for HCoV LRI in our study and we used an ISI cutoff of ≥ 5 instead of analyzing ISI as a continuous variable as Ogimi et al. [16]. We also reported a higher mortality rate due to HCoV LRI and respiratory failure when compared to Pinana et al. [18] (17% vs. 7.4%), but lower to that reported by Ogimi et al. [6] (17% for 30‐day mortality and 30% for 90‐day mortality).

HCoV LRI has been associated with poor outcomes, besides mortality, in both immunocompetent and immunocompromised patients. HCT recipients and patients with hematologic malignancies with HCoV infections have high rates of mechanical ventilation leading to longer hospital and ICU stays [6]. We found similar poor outcomes in our cohort with higher hospital and ICU admission rates and higher mechanical ventilation rates. These findings underscore the unmet need when it comes to direct‐antiviral therapy for vulnerable patient population.

In this current study, not all of the ISI components were associated with HCoV LRI; instead, the combination of these risk factors with an ISI of ≥ 5 was a predictor of LRI, especially when it comes to its negative predictive value (87%). This makes the ISI a versatile tool that can be used to stratify HCT recipients based on the combination of multiple risk factors of worse outcomes and with any type of respiratory viral infections for which sparse data are available, such as SARS‐CoV‐2. Prospective studies validating the use of the ISI to predict progression to LRI is needed to verify its clinical utility.

Seasonal HCoV was reported to be a common cause of acute respiratory viral infections in both children and adults prior to the spread of SARS‐CoV‐2 [19]. HCoV infections can lead to severe infections in older patients with underlying cardiopulmonary disease or immunocompromised conditions [5, 20]. In comparison, patients with COVID‐19 presented with a wide spectrum of illness [7, 8]. In retrospective studies of cancer patients with COVID‐19, risk factors for hospitalizations included non‐White patients, patients with hematologic malignancies, chronic lymphopenia, steroids use, cardiac comorbidities, and treatment with immune checkpoint inhibitors [21, 22]. Among HCT recipients with COVID‐19, attributable mortality is up to 17% and 14% in allogeneic and autologous HCT recipients, respectively, based on pooled analysis [8]. In comparison, in our cohort of HCT recipients with HCoV infections, 30‐day mortality rate was only 4% overall, with an in‐hospital mortality rate of 11% (6 deaths from 56 admissions).

Our study has few limitations. It is a retrospective study, which makes it difficult to draw exact causal relationships between HCoV LRI and the studied risk factors. There was a limited number of LRI episodes and few cases of documented progression to LRI with subsequent wide confidence intervals noted in our multivariate analysis for LRI in HCoV infected patients. Due to limited number of deaths, we did not perform further analysis to identify independent risk factors for mortality. In addition, this study from a large comprehensive single cancer center may limit the generalizability of the results. However, our results confirm those reported by other centers.

In summary, we identified multiple risk factors for HCoV LRI in HCT recipients and the use of the ISI may determine the patients at risk who may benefit the most from targeted therapy.

## Conflicts of Interest

Fareed Khawaja: Research funding paid to his institution from MERCK, Eurofins Viracor, and Symbio Pharma. May Daher and the University of Texas MD Anderson Cancer Center have an institutional financial conflict of interest with Takeda Pharmaceutical. May Daher participates on the scientific advisory board of Cellsbin and Aurigene. Roy F. Chemaly: Consultant/Speaker/Advisor for ADMA Biologics, Janssen, Merck/MSD, Partner Therapeutics, Takeda, Shinogi, AiCuris, Roche/Genentech, Astellas, Tether, Oxford Immunotec, Karius, Moderna, Pfizer, Invivyd, Eurofins‐Viracor, and Ansun Pharmaceuticals and received Research grants paid to his institution from Merck/MSD, Karius, AiCuris, Ansun Pharmaceuticals, Takeda, Genentech, Oxford Immunotec, and Eurofins‐Viracor. The other authors declare no conflicts of interest.

## Supporting information




**Supplemental Table 1**: Immunodeficiency index components.
**Supplemental Figure 1**: Seasonal Distribution of Human Coronavirus Serotypes among Hematopoietic Cell Transplant Recipients between September 2015 and August 2017.
**Supplemental Figure 2**: Percentage of Lower (LRI) and Upper respiratory Tract Infections (URI) stratified by Human Coronavirus Serotypes in Hematopoietic Cell Transplant Recipients.
**Supplemental Figure 3**: ROC Curve to Determine the Optimal Immunodeficiency Scoring Index Cut‐Off to Predict Lower Respiratory Infection in Hematopoietic Cell Transplant Recipients.
