# Supplementary material for: Burden of Seasonal Human Coronavirus Infections in Hematopoietic Cell Transplant Recipients
Source: Transpl Infect Dis. 2025 Aug 21;27(5):e70094. doi: 10.1111/tid.70094 (PMC12519910; doi:10.1111/tid.70094)

**Supplemental material:**

**Tables and figures:**

**Supplemental table 1. Immunodeficiency index components**

| **Component** | **Assigned score** |
| --- | --- |
| Absolute neutrophil count of less than 500 cell/µL | **3** |
| Absolute lymphocyte count of less than 200 cell/µL | **3** |
| Age of 40 years or greater | **2** |
| The use of a myeloablative conditioning regimen | **1** |
| Any graft versus host disease | **1** |
| Corticosteroids used within 30 days of respiratory viral infection | **1** |
| Recent (within 30 days of infection) or pre engraftment allogeneic HCT | **1** |

*Abbreviations HCT: Hematopoietic cell transplantation*

**Supplemental figure 1. Seasonal Distribution of Human Coronavirus Serotypes among Hematopoietic Cell Transplant Recipients between September 2015 and August 2017**

**Supplemental figure 2. Percentage of Lower (LRI) and Upper respiratory Tract Infections (URI) stratified by Human Coronavirus Serotypes in Hematopoietic Cell Transplant Recipients**

**Supplemental figure 3. ROC Curve to Determine the Optimal Immunodeficiency Scoring Index Cut-Off to Predict Lower Respiratory Infection in Hematopoietic Cell Transplant Recipients**


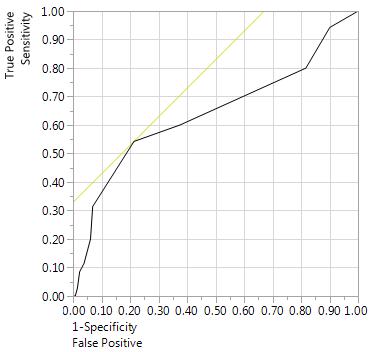

Supplement: Supplementary file 1 — Supplemental Table 1: Immunodeficiency index components. Supplemental Figure 1: Seasonal Distribution of Human Coronavirus Serotypes among Hematopoietic Cell Transplant Recipients between September 2015 and August 2017. Supplemental Figure 2: Percentage of Lower (LRI) and Upper respiratory Tract Infections (URI) stratified by Human Coronavirus Serotypes in Hematopoietic Cell Transplant Recipients. Supplemental Figure 3: ROC Curve to Determine the Optimal Immunodeficiency Scoring Index Cut‐Off to Predict Lower Respiratory Infection in Hematopoietic Cell Transplant Recipients. [file TID-27-e70094-s001.docx]
